# *N*-acetylcysteine prevents olanzapine-induced oxidative stress in mHypoA-59 hypothalamic neurons

**DOI:** 10.1038/s41598-020-75356-3

**Published:** 2020-11-05

**Authors:** Zehra Boz, Minmin Hu, Yinghua Yu, Xu-Feng Huang

**Affiliations:** 1 Illawarra Health and Medical Research Institute and School of Medicine, University of Wollongong, Wollongong, NSW 2522 Australia; 2 Jiangsu Key Laboratory of Immunity and Metabolism, Xuzhou Medical University, Xuzhou, 221004 Jiangsu China

**Keywords:** Cellular neuroscience, Molecular neuroscience, Neuronal physiology

## Abstract

Olanzapine is a second-generation antipsychotic (AP) drug commonly prescribed for the treatment of schizophrenia. Recently, olanzapine has been found to cause brain tissue volume loss in rodent and primate studies; however, the underlying mechanism remains unknown. Abnormal autophagy and oxidative stress have been implicated to have a role in AP-induced neurodegeneration, while *N*-acetylcysteine (NAC) is a potent antioxidant, shown to be beneficial in the treatment of schizophrenia. Here, we investigate the role of olanzapine and NAC on cell viability, oxidative stress, mitochondrial mass and mitophagy in hypothalamic cells. Firstly, cell viability was assessed in mHypoA-59 and mHypoA NPY/GFP cells using an MTS assay and flow cytometric analyses. Olanzapine treated mHypoA-59 cells were then assessed for mitophagy markers and oxidative stress; including quantification of lysosomes, autophagosomes, LC3B-II, p62, superoxide anion (O_2_^–^) and mitochondrial mass. NAC (10 mM) was used to reverse the effects of olanzapine (100 µM) on O_2_^−^, mitochondrial mass and LC3B-II. We found that olanzapine significantly impacted cell viability in mHypoA-59 hypothalamic cells in a dose and time-dependent manner. Olanzapine inhibited mitophagy, instigated oxidative stress and prompted mitochondrial abnormalities. NAC was able to mitigate olanzapine-induced effects. These findings suggest that high doses of olanzapine may cause neurotoxicity of hypothalamic neurons via increased production of reactive oxygen species (ROS), mitochondrial damage and mitophagy inhibition. This could in part explain data suggesting that APs may reduce brain volume.

## Introduction

Olanzapine is a second-generation obesogenic antipsychotic (AP) drug prescribed for the treatment of schizophrenia, bipolar mania and depression among other mental illnesses^1^. It has been well documented that olanzapine has the potential to cause neurodegeneration^2^. For example, meta-analyses report global reduction in brain size, grey matter reduction, enlargement of ventricles, cortical thinning and subcortical thickening in patients taking AP medication^3–5^. Preclinical studies demonstrate similar findings and provide a better understanding of isolated drug effect without the interplay of disease^6^. Recently, olanzapine has been reported to cause brain tissue volume loss and cortical thinning in animal studies^7,8^. Few reports suggest that oxidative stress, mitochondrial damage and autophagy inhibition have a role in AP-induced cortical thinning^9,10^. However, the mechanism underlying AP-induced neurodegeneration remains largely ambiguous.

Reactive oxygen species (ROS) more commonly referred to as “free radicals” are mainly produced in the mitochondria. ROS have unpaired electrons in an oxygen atom making them highly unstable; hence, they react quickly with surrounding molecules in order to pair their electrons^11^. This usually leads to a chain reaction which activates numerous signalling pathways. When ROS are transiently produced, they act as signal molecules^12^. However, when this balance is altered, excessive ROS will cause oxidative stress that may be lethal to the cell^12^. In this case, mitophagy—a specific type of autophagy – will attempt to maintain the cells integrity and stability by “recycling” damaged and long-lived mitochondria. If mitochondria are not disposed of in a timely manner they release caspases which can initiate apoptosis (programmed cell death)^13^. Autophagy is a vital cellular energy generating process whereby outdated or damaged organelles such as mitochondria are degraded for energy re-consumption. This process improves the efficiency and longevity of the cell. Briefly, an autophagosome containing membrane proteins LC3B and p62 will engulf all “recyclable” units and carry them to a lysosome. The autophagosome and lysosome will fuse to form an autophagolysosome. Here, the damaged cellular units and p62 are degraded by acidic vesicles in the lysosome and are used as an energy source. It has previously been noted that schizophrenia patients have a marked increase in oxidative stress markers. For example, thiobarbituric acid-reactive substances (TBARS) and malondiadehyde (MDA) levels in patients are significantly increased^14,15^. Chronic olanzapine treatment also increases ROS production and MDA in SHSY5Y cells^9,16–18^. Furthermore, autophagy has a key role in the pathophysiology of schizophrenia. For example, Beclin1 (an essential autophagy protein) has been found to be decreased by 40% in post-mortem hippocampal tissue of schizophrenia patients^19^. Moreover, post-mortem analysis of brain tissue confirm increased mitophagy in schizophrenia patients^20,21^. Interestingly, clinically approved olanzapine, haloperidol, clozapine and sertindole have been found to induce autophagy in neuronal cells^22–24^. Therefore, it is possible that the oxidative stress and autophagy observed in patients is caused by AP medication.

Most literature supporting AP-induced neurodegeneration focuses on the frontal and temporal lobes, however, a 10% reduction in whole brain volume has been reported in animal models with a strong focus on grey matter reductions^2^. The primary function of the hypothalamus is metabolic regulation, yet, the hypothalamus is also grey matter^25^. Olanzapine is notorious for causing weight-gain and other metabolic side-effects^26^. It is possible this is related to the neurodegeneration of grey matter in the hypothalamus. Previous studies investigating the role of APs on the autophagy pathway have used SH-SY5Y cells or primary neurons^24^. Therefore our study is novel in using hypothalamic neuronal cells to investigate the role of mitophagy in AP-induced neurodegeneration.

*N*-acetylcysteine (NAC) is a potent antioxidant derived from the amino acid L-cysteine and is used for the treatment of various conditions such as schizophrenia, amyotrophic lateral sclerosis, bipolar disorder, Alzheimer’s disease and various cancers^27^. We hypothesise that NAC may alleviate olanzapine-induced effects on neurodegeneration via countering oxidative stress.

In this study we aimed to investigate the underlying mechanism of olanzapine-induced neurodegeneration, primarily focusing on the role of oxidative stress, mitochondrial damage, and mitophagy in mHypoA-59 hypothalamic neurons. We also tested the hypothesis that antioxidant NAC would alleviate any observed effects.

## Materials and methods

### Experimental design—cell culture and treatments

The mHypoA-59 hypothalamic neuronal cell line (Cedarlane, CLU468) and mHypoA NPY/GFP (Cedarlane, CLU499) were grown at 37 °C in a humidified 5% CO_2_ incubator in Dulbecco’s Modified Eagle Medium (Sigma Aldrich, DMEM-5796) supplemented with or without 10% FBS (Bovogen Biologicals, SFBS-F) and 1% penicillin/streptomycin (ThermoFisher, 15140122). Trypsinized cells were seeded (1.5 × 10^4^) in CELLSTAR Greiner 96-well flat-bottom microplates for cell viability and FlexStation fluorescence readings, Greiner 24-well plates (1.2 × 10^5^) for imaging and flow cytometric analysis, or (0.8 × 10^6^) Greiner 6 well plates for immunoblotting. Cells rested for 24hrs before treatment with either olanzapine (Sigma-Aldrich, O1141) in the presence or absence of *N*-acetylcysteine (Sigma-Aldrich, A7250) as described in results.

### MTS assay & cell viability

Cell viability was assessed using the CellTiter 96 Aqueous One Solution Cell Proliferation Assay and absorbance was measured at 490 nm. Cell viability was confirmed using flow cytometric analysis to outline live cell population while a live-cell imager was used to illustrate these results.

### Acridine orange staining

Acidic vesicles were quantified using acridine orange (AO) (Sigma-Aldrich, 318337); a pH sensitive dye used to detect lysosomal activity. We used flow cytometry or fluorescent microscopy to detect FL1/orange-red vs. FL3/green fluorescence.

### Cyto-ID staining

Pre-autophagosomes, autophagosomes and autophagolysosomes were measured by staining with the Cyto-ID Autophagy detection kit (ENZO, 51031) followed by flow cytometric analysis.

### ROS and mitochondrial stress

MitoSOX Red Mitochondrial Superoxide Indicator (ThermoFisher Scientific, M36008) was used to detect mitochondrial ROS, while, MitoTracker Green FM (ThermoFisher Scientific, M7514) was used to determine mitochondrial mass. Cells were stained with the given dyes followed by flow cytometric analyses and fluorescent imaging.

### Immunoblot analysis

Cells were treated for 24hrs, harvested with lysis buffer and protein concentration was determined using DC Protein Assay Kit (Bio-Rad, 5000111). Samples were heated at 95 °C in Laemmli buffer, loaded for fractionation to 10% SDS-PAGE gels (Bio-Rad, 4561033), and then transferred into Immuno-Blot PVDF membranes (Bio-Rad, 1620177). 5% skim milk in TBST was used as blocking buffer. Membranes were incubated overnight at 4◦C with the following antibodies; LC3B (Cell Signalling Technology, #2775), and p62 (Cell Signalling Technology, 5114) in TBST containing 1% milk. Anti-rabbit IgG conjugated with horseradish peroxidase was used as the secondary antibody (Santa Cruz Biotechnology). We used ECL detection reagents (Sigma-Aldrich, RPN3004) and captured high resolution images with Amersham Gel Imager (GE Healthcare life Sciences).

### Statistical analysis

Statistical analysis was performed with the SPSS program (version 21; Chicago, IL, United States). One-way analysis of variance (ANOVA) followed by Tukey’s post hoc test was used for multiple comparisons for all data as there were only two factors at any given time. This includes data for cell viability, AO, MitoSOX and MitoTracker flow cytometric analysis as well as LC3B-II and p62 immunoblotting. Data were expressed as mean ± SEM, and *p* < 0.05 was considered statistically significant.

### Significance statement

Olanzapine is one of the most commonly prescribed second-generation antipsychotic drugs. Notoriously known for metabolic side-effects such as weight gain and diabetes, olanzapine has also been demonstrated to cause neurodegeneration. However, there is limited mechanistic knowledge surrounding this area. Here, for the first time, we demonstrate that olanzapine causes cell death in mHypoA-59 hypothalamic neurons via increased oxidative stress and mitochondrial damage. Furthermore, the antioxidant *N*-acetylcysteine is able to reverse these effects. This study provides a base-line in understanding the mechanism underlying olanzapine-induced neurodegeneration and proposes a potential treatment outlet for these severe side-effects.

## Results

### Olanzapine may cause neuronal toxicity in a dose and time-dependent manner in hypothalamic neurons

**Figure 1:**
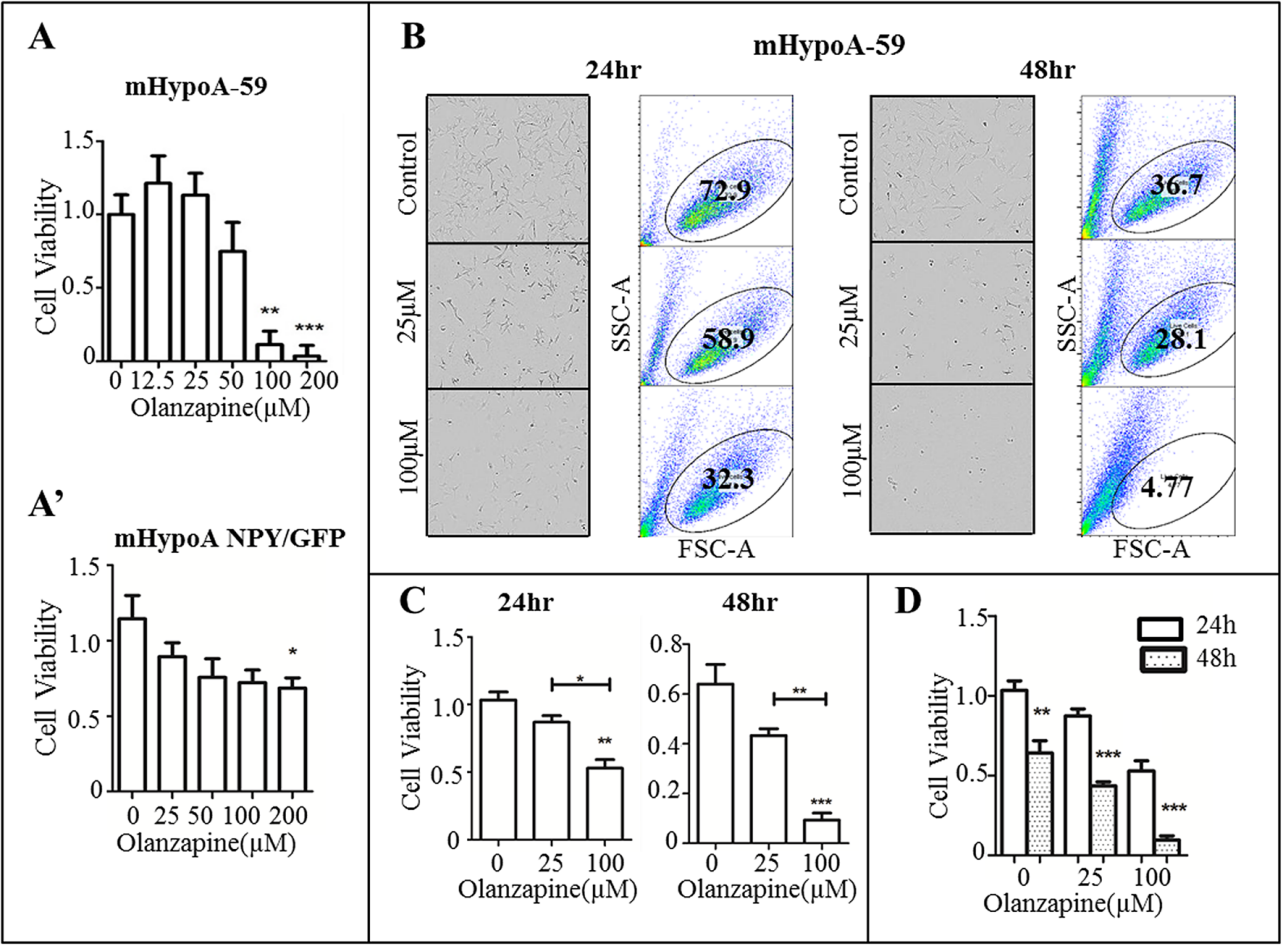
Olanzapine induced neuronal toxicity in a dose and time-dependent manner. (**A**) mHypoA-59 neurons and (**A’**) mHypoA NPY/GFP neurons were incubated with various concentrations of olanzapine for 24hrs and cell viability was assessed using an MTS assay (**B–D**) mHypoA-59 neurons were then treated with either 25 or 100 µM of olanzapine for 24 or 48hrs, cells were imaged using a live-cell imager and quantified by flow cytometry. (*p < 0.05, ** *p* < 0.01, ***p < 0.001 compared to the control group, and compared to 24 hr in Fig. 1D. Data is presented as mean ± SEM).

Hypothalamic neurons have been used in vitro to investigate central nervous system disorders such as obesity, stress and metabolic conditions^28,29^. An MTS assay was used to determine cell viability and optimum working concentrations for both mHypoA-59 neurons and mHypoA NPY/GFP after 24 h of olanzapine treatment. mHypoA-59 neurons were susceptible to cell toxicity at concentrations ≥ 100 µM after 24 h (Fig. 1A; *p* = 0.001 , F_(5, 47)_ = 12.455). In mHypoA NPY/GFP neurons, olanzapine significantly decreased cell viability at 200 µM (Fig. 1A’, *p* = 0.04, F_(4, 38)_ = 2.918).

Additionally, live cell images and flow cytometric quantification confirmed neurotoxicity of mHypoA-59 neurons treated with 100 µM of olanzapine at both 24 and 48 h. 100 µM of olanzapine decreased cell viability at 24hrs (Fig. 1B,C, p = 0.008. F_(2, 8)_ = 11.928) and this was further exaggerated at 48hrs (Fig. 1D, p = 0.001. F_(2, 8)_ = 29.858). No change was observed at 25 µM.

### Olanzapine causes lysosome and autophagosome accumulation in mHypoA-59 cells

**Figure 2:**
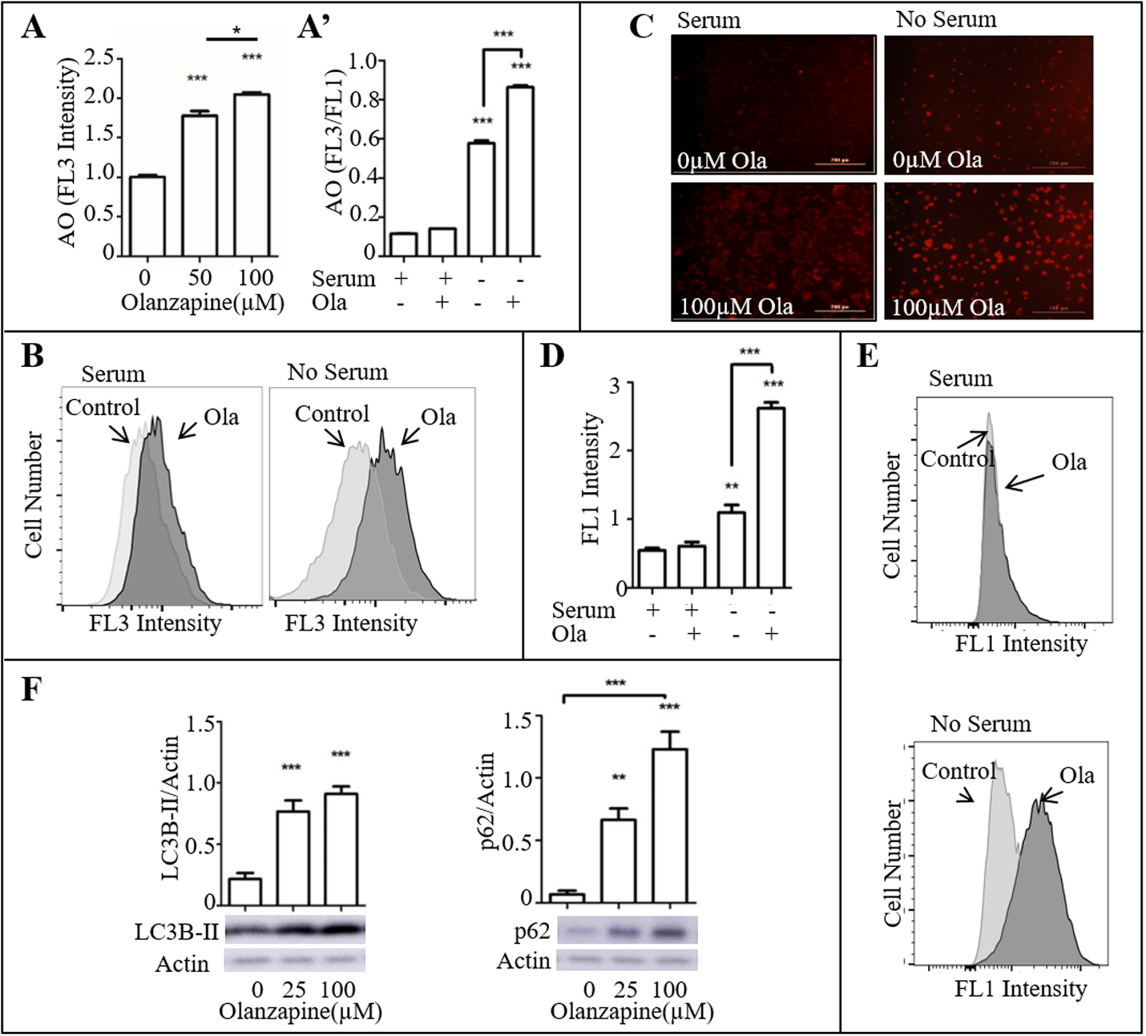
Olanzapine caused lysosome and autophagosome accumulation in mHypoA-59 neurons in a dose-dependent manner under serum-starved conditions. (**A–C**) mHypoA-59 neurons were incubated for 24hrs with and without 100 µM olanzapine under serum and serum-starved conditions. After staining with acridine orange (AO), quantification of acidic vesicles were determined as an increase in red (FL3) vs. green (FL1) using flow cytometry (**A**,**B**)**.** Flow cytometric assessment of FL3 intensity (**C**)**.** Respective images of A-B using a live-cell imager. (**D,E**) mHypoA-59 neurons were incubated for 24 h with or without 100 µM olanzapine under serum and serum-starved conditions. After staining with Cyto-ID, autophagosomes were quantified measuring an increase in green fluorescence (FL1) using flow cytometry (** *p* < 0.01, ***p < 0.001 compared to the serum only group). (**F**) mHypoA-59 neurons were incubated for 24hrs with and without 100 µM olanzapine under serum-starved conditions, LC3B-II and p62 were assessed via immunoblotting (* *p* < 0.05, ***p < 0.001 compared to the serum only group, data is presented as mean ± SEM) Abbreviations: Ola: Olanzapine.

Excessive or aberrant autophagy can lead to neurodegeneration and cell death^30,31^. Thus, we examined the efficiency of the autophagy process under olanzapine treatment. AO is a fluorescent dye commonly used to detect lysosomal activity. This dye usually fluoresces green and becomes orange-red upon interaction and protonation with the acidic vesicles found in lysosomes. Flow cytometric analysis of AO stained mHypoA-59 neurons demonstrated that olanzapine increases the amount of red-marked acidic vesicles in the lysosomes under serum-starved conditions (Fig. 2A’, the representative histogram is illustrated in Fig. 2B, p < 0.001, F_(3, 11)_ = 138.872). The observed effect of olanzapine was also dose-dependent (Fig. 2A, p < 0.001. F_(2, 8)_ = 51.886). Live cell imaging analysis with the Lionheart FX confirmed an increase in AO stained acidic vesicles (Fig. 2C).

Cyto-ID stains autophagic vacuoles; which includes, pre-autophagosomes, autophagosomes and autophagolysosomes. Hence, we used Cyto-ID to determine the level of autophagic components found after olanzapine exposure in mHypoA-59 neurons. Flow cytometric analysis of Cyto-ID stained mHypoA-59 neurons demonstrated that olanzapine increases the amount of autophagic vacuoles in serum-starved conditions (Fig. 2D, the representative histogram is illustrated in Fig. 2E, p < 0.001, F_(3, 11)_ = 148.547).

LC3B-II and p62 are key proteins in the autophagy process and are commonly used as markers for autophagy inhibition or completion. The LC3B and p62 proteins are required to form the membrane of the autophagosome. While, LC3B is accumulated with autophagy upregulation, p62 is degraded upon completion of autophagy. Interestingly, western blot analysis revealed a significant increase in both LC3B-II and p62 proteins indicating an aberrant autophagy process (Fig. 2F). LC3B-II showed a significant increase at both 25 µM (*p* < 0.001, F_(2,17)_ = 28.449) and 100 µM (*p* < 0.001). P62 also showed a significant increase at both 25 µM (*p* = 0.002, F_(2, 17)_ = 34.778) and 100 µM (*p* < 0.001).

### Olanzapine induces oxidative stress and mitochondrial dysfunction in mHypoA-59 cells

**Figure 3:**
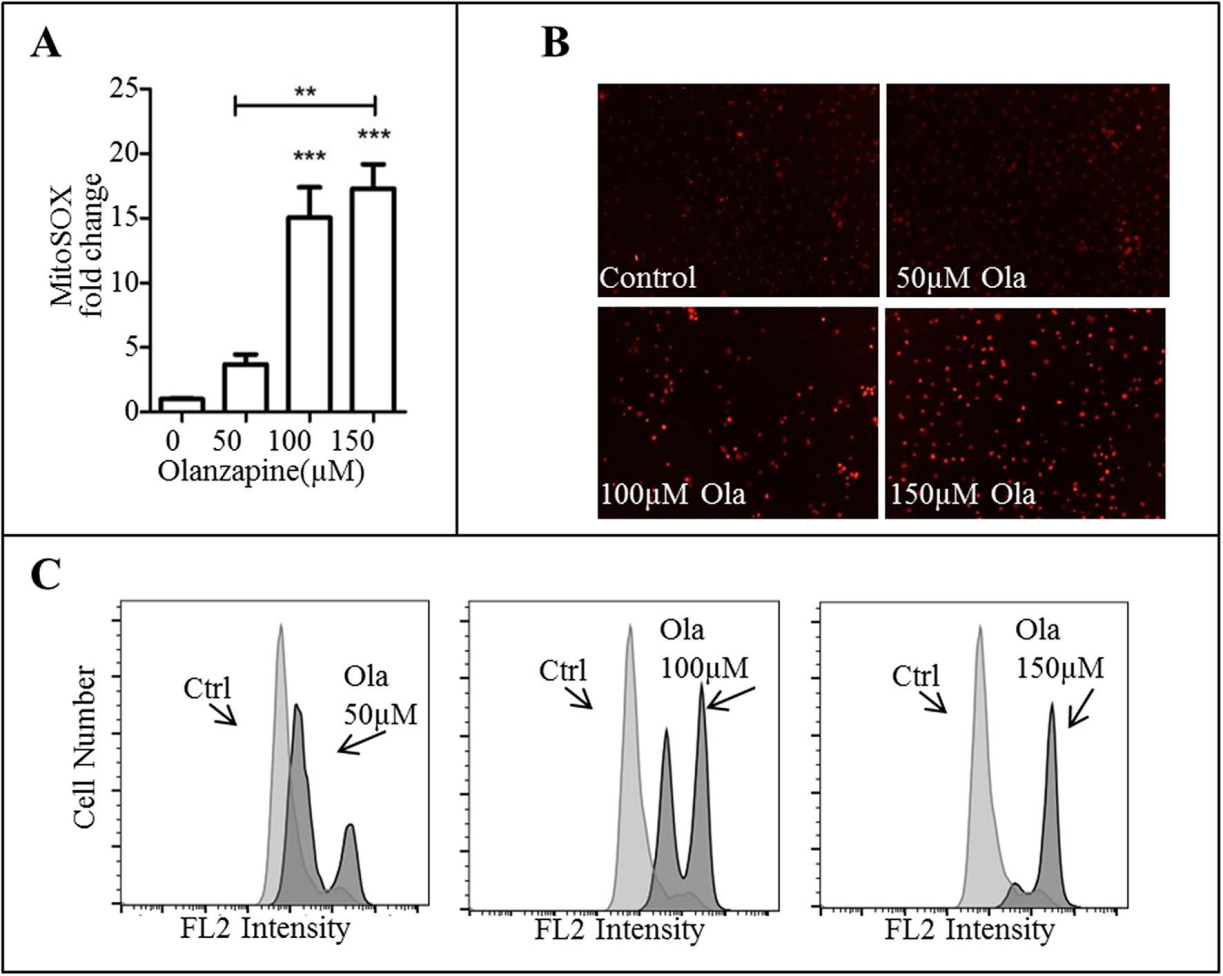
Olanzapine increased MitoSOX – superoxide anion (O_2_^−^) in mHypoA-59 neurons (**A–C**) mHypoA-59 neurons were incubated for 24hrs with either 50 µM, 100 µM or 150 µM of olanzapine and stained with MitoSOX for O_2_^−^. (**A**) fold change of MitoSOX quantified by flow cytometry (**B**) live cell images (**C**) fluorescence intensity via flow cytometry. (*p < 0.05, ** *p* < 0.01, ***p < 0.001 compared to the control group – the control group was stained with MitoSOX however was not treated with olanzapine. Data is presented as mean ± SEM).

Aberrant autophagy is commonly related to oxidative stress and mitochondrial damage. To investigate this pathway further, we used MitoSOX to measure ROS production. Flow cytometric analysis of MitoSOX stained mHypoA-59 cells revealed a significant increase of mitochondrial ROS (O_2_^−^) in a dose-dependent manner (Fig. 3A, representative histogram is illustrated in Fig. 3C, p = 0.001 at 100 µM and *p* < 0.001 at 150 µM, F_(3, 11)_ = 26.887). This increase was also confirmed with live cell images (Fig. 3B).

**Figure 4:**
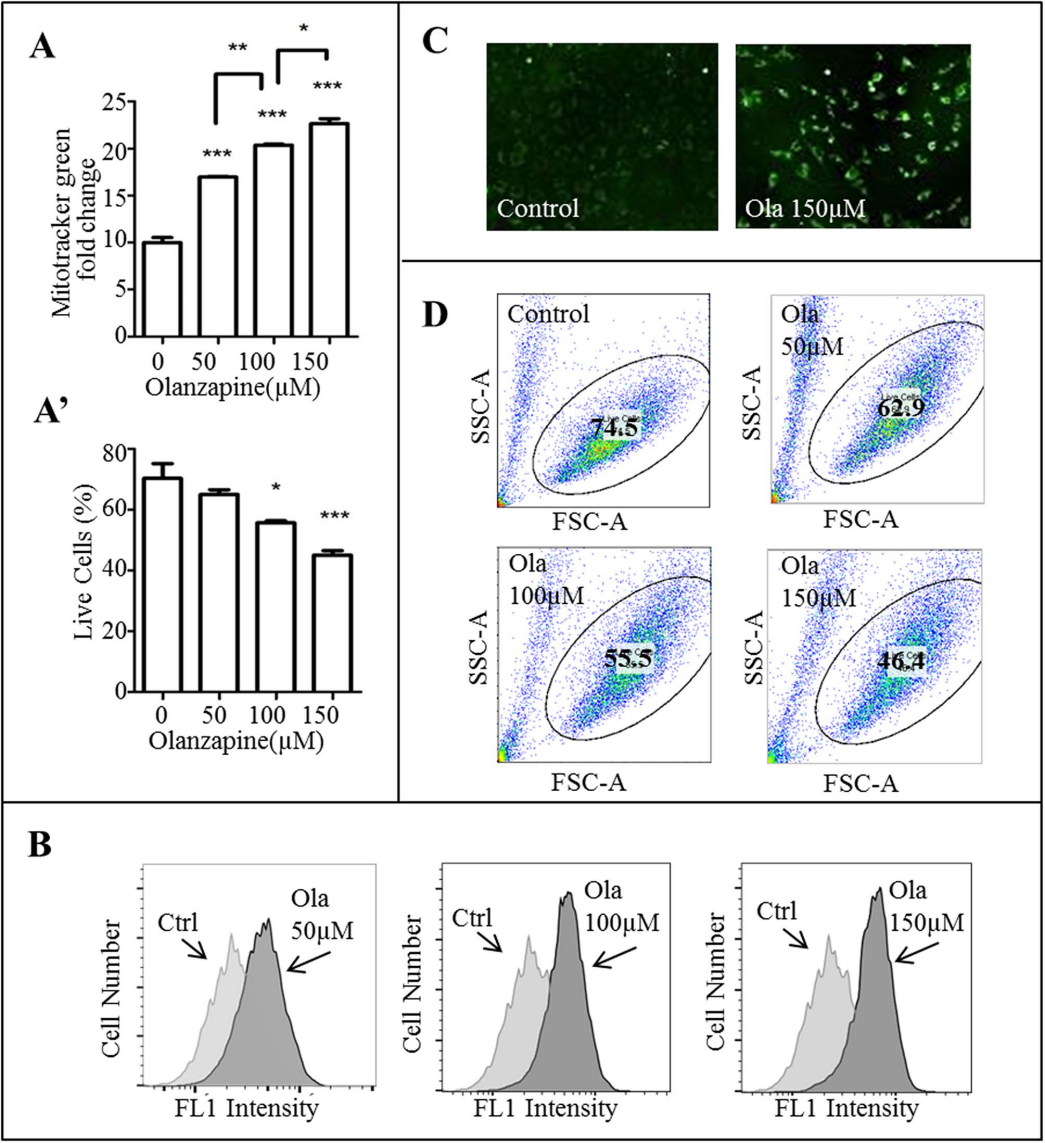
Olanzapine increased mitochondrial mass but decreased the percentage of live cells in mHypoA-59 neurons over 24hrs. (**A–C**) mHypoA-59 neurons were incubated for 24hrs with either 50 µM, 100 µM or 150 µM of olanzapine and stained with MitoTracker for mitochondrial mass. (**A**) Fold change of mitochondrial mass and live cell population percentage quantified by flow cytometry. (**B**) Fluorescence intensity via flow cytometry. (**C**) mHypoA-59 neurons imaged with a live cell imager. (**D**) Live cell population at various concentrations of olanzapine (*p < 0.05, ** *p* < 0.01, ***p < 0.001 compared to the control group – the control group was stained with MitoTracker however was not treated with olanzapine. Data is presented as mean ± SEM).

Further examination of mitochondrial stress revealed an abnormality in mitochondrial mass. Flow cytometric analysis of MitoTracker stained mHypoA-59 cells showed that olanzapine significantly increased mitochondrial mass in a dose-dependent manner (Fig. 4A, representative histogram is illustrated in Fig. 4B, p < 0.001, F_(3, 11)_ = 213.787 for all concentrations). Live cell imaging with the incucyte ZOOM confirmed an increase in green fluorescent mitochondria (Fig. 4C). This increase in mitochondrial mass correlated with a decline in live cell population (Fig. 4A’, representative scatter plot is illustrated in Fig. 4D, p = 0.021 at 100 µM and *p* = 0.001 at 150 µM, F_(3, 11)_ = 16.929). Given the autophagy pathway was inhibited; it is possible that increased mitochondrial mass indicates aberrant mitophagy and thereby causes cell death.

### ROS inhibitor NAC reversed olanzapine-induced oxidative stress

**Figure 5:**
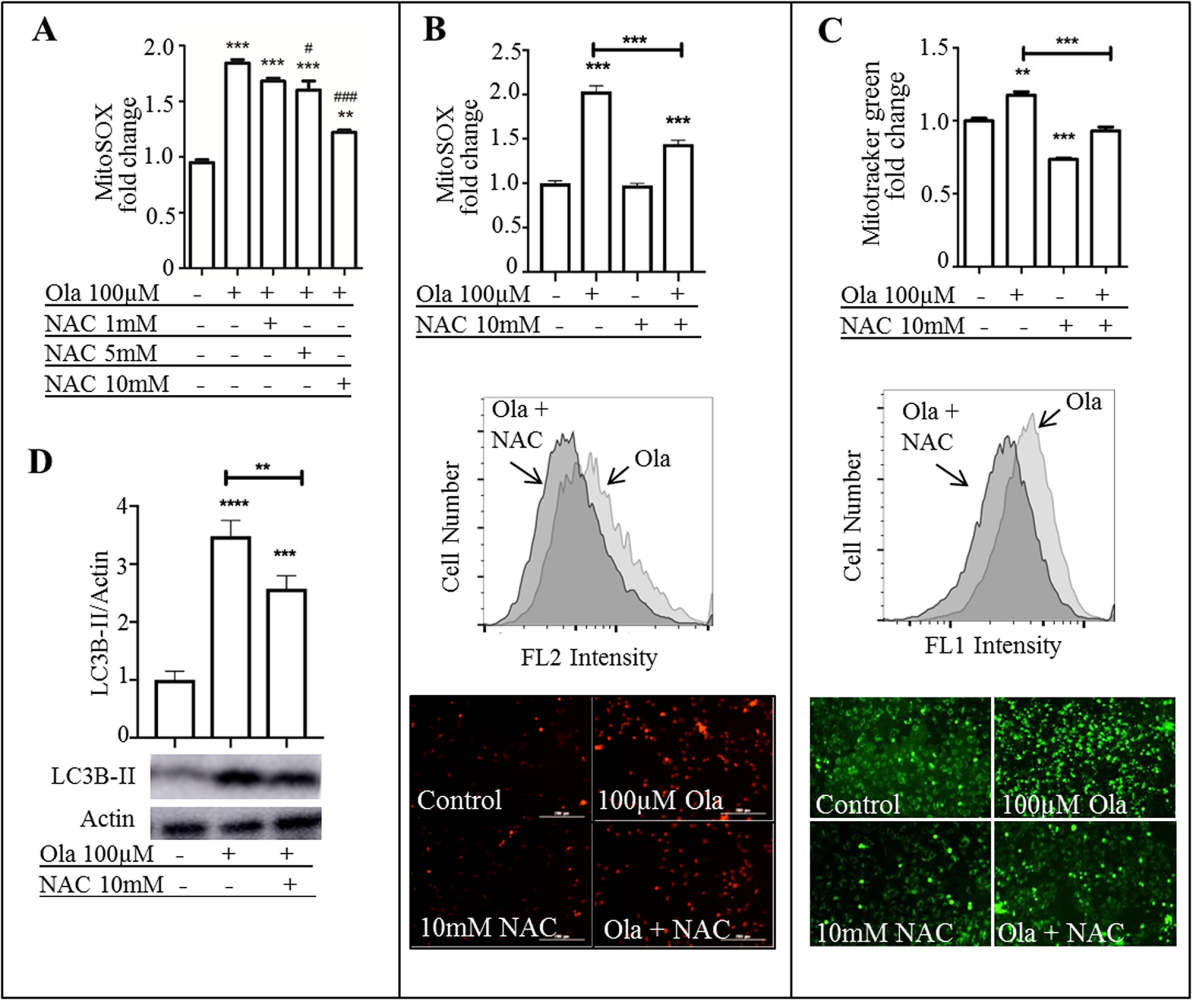
ROS inhibitor NAC reversed olanzapine-induced oxidative stress and mitochondrial mass. (**A**) 10 mM NAC dosage was established via pretreating cells with 1–10 mM for 30mins before adding 100 µM olanzapine for 24hrs. Neurons were then stained with MitoSOX and fluorescence intensity was quantified via flow cytometry (**B,C**) mHypoA-59 neurons were incubated for 24hrs with either 100 µM olanzapine, 10 mM NAC or both under serum-starved conditions. (**B**) neurons were quantified via flow cytometry and imaged following MitoSOX staining. (**C**) neurons were quantified via flow cytometry and imaged following MitoTracker staining. (**D**) LC3B-II was quantified after immunoblotting. Abbreviations: Ola: Olanzapine, NAC: N-acetyl cysteine (*p < 0.05, ** *p* < 0.01, ***p < 0.001, **** < 0.0001 compared to the control group, # *p* < 0.05, ###* p* < 0.001 compared to the olanzapine group).

Among others, one route to instigate autophagy is oxidative stress. We found that free radicals (O_2_^−^) are accumulated upon olanzapine treatment of hypothalamic neurons. N-Acetyl Cysteine (NAC) is commonly used for ROS inhibition. Therefore, NAC was used to ameliorate the oxidative and mitochondrial damage inflicted by olanzapine. Ranges between 500 µM and 10 mM NAC have been used in previous literature pertaining to neuronal cell culture^32–37^. Therefore, we performed an initial experiment with various doses of NAC (1 mM-10 mM) to reverse olanzapine-induced ROS detected using MitoSOX(Fig. 5A). 1 mM NAC had no effect while 5 mM (*p* < 0.05) and 10 mM (*p* < 0.001) reduced ROS in olanzapine treated cells. Therefore, 10 mM NAC was used for the remainder of the experiments. Accordingly, 10 mM NAC significantly decreased O_2_^−^ levels (Fig. 5B, p < 0.001, F_(3, 11)_ = 142.996) and mitochondrial mass (Fig. 5C. *p* < 0.001, F_(3, 11)_ = 84.548) compared to the olanzapine only treated group, when applied to neurons 30 min prior to olanzapine treatment. Correspondingly, LC3B-II levels significantly decreased with 10 mM of NAC treatment compared to the olanzapine only treated group (Fig. 5D, p = 0.006, F_(2, 8)_ = 97.369).

## Discussion

Here, using cellular techniques, we have demonstrated that (i) olanzapine induces oxidative stress, inhibits mitophagy and causes apoptosis in mHypoA-59 neuronal cells; (ii) NAC is able to reverse olanzapine-induced oxidative stress and cellular damage.

Olanzapine is generally prescribed at a range between 5–20 mg/day and dosage is usually increased in 5 mg intervals on a fortnightly basis. However, more and more frequently case reports confirm prescriptions above the recommended range. Between 1997 and 2006, the percentage of patients prescribed olanzapine above the licensed range of 20 mg/day increased from 16.2% to over 50%^38^. This is particularly the case for treatment resistant patients and those presenting with acute or high levels of psychopathology^39^. A study conducted between nine psychiatric centres in Denmark, concluded that 80% of patients were administered between 50 and 80 mg/day, the maximum dose being 160 mg/day^40^. Another study examined 58 post-mortem blood specimens and found a mean olanzapine concentration of 358 ng/ml with a range of 10 to 5200 ng/ml^41^. Since serum levels should not exceed 100 ng/ml, it is clear that patients are taking more than what have previously been reported^42^. Given these accounts from clinical practice and post-mortem blood serum levels it is not uncommon that doses of olanzapine higher than recommended are prescribed to patients.

Therapeutic serum levels of olanzapine are reported to be between 0.1–0.3 µM^43,44^; however, these serum levels are based on patients receiving only 5–20 mg/day. Interestingly, post-mortem samples reveal an average of 1.1 µM olanzapine blood concentration, ranging up to 16 µM^41^. It is known that antipsychotics accumulate in brain tissue at 25–30 folds higher than serum levels^45,46^. Clinical studies predict a micro molar concentration in the brain^47^. Although in vitro doses are not exactly comparable to doses in vivo, the olanzapine concentration in this study ranging between 25 and 150 µM used for neuronal culture may mimic the micro molar concentration of olanzapine predicted in the CNS of patients.

This present study demonstrates that olanzapine induces cell death in mHypoA-59 hypothalamic neurons in a dose and time-dependent manner, but not mHypoA NPY/GFP cells. Interestingly, previous studies extensively report the neuroprotective nature of olanzapine against various agents. For example, olanzapine (1 mg/kg) prevents methamphetamine-induced apoptosis in the medial prefrontal cortex of rats injected daily for 5 days^48^. Another study reports olanzapine (80–120 µM) neuroprotection of glutamine and serum deprived PC12 cells after 72 h^49^. Similarly, the neuroprotective nature of olanzapine (100–200 µM) is reported against hydrogen peroxide toxicity in PC12 cells after 24hrs of treatment^48–50^. Olanzapine is also reported to increase cell survival in PC12 cell cultures, SH-SY5Y cells, and 3T3 preadipocytes^49,51^. Conversely, the neurotoxicity of olanzapine is not well documented in cell culture studies, particularly hypothalamic neurons. However, one study reports the neurotoxic nature of olanzapine (100 µM) in SH-SY5Y cells^9^. Interestingly, haloperidol and clozapine (10–25 µM) treated primary rat neurons, significantly decreases cell viability of neurons in a dose and time-dependent manner^22^.

Comparable to the results found in mHypoA NPY/GFP cells here, Kowalchuk et. al. report that neither 24 h olanzapine treatment at 20 µM or 100 µM effected cell viability in rHypoE-19 and rHypoE-46 hypothalamic cells^29^. The variance in cell viability observed between hypothalamic cell lines could be due to higher olanzapine tolerability of mHypoA NPY/GFP cells compared to mHypoA-59 cells. Previously, differential responses have been observed in similar cell lines in reaction to hormones and other compounds^52^. This can be attributed to the differing origin of the cell lines, differential receptor profiles or other physical properties such as the addition of a GFP as seen here.

Nonetheless, in vivo, there are substantial reports to suggest the neurotoxic nature of olanzapine as well as other antipsychotics. For example, Vernon et al.^7^ report brain volume reductions of 10%, primarily of grey matter, in rats infused with clinical levels of either olanzapine or haloperidol for 8 weeks compared to untreated controls. Another study in monkeys also reveals an 8–11% loss of brain volume after oral administration of olanzapine or haloperidol for a period of 17–27 months^53,54^. Here we show that olanzapine can cause hypothalamic toxicity in neurons, which form the bases of hypothalamic grey matter. Olanzapine causes severe metabolic side effects and it is possible that these are related to hypothalamic toxicity and neurodegeneration. However, a future study confirming this hypothesis in vivo is necessary for a better understanding of the effects of APs on grey matter.

Taken together, it is possible that olanzapine elicits neuroprotective properties in neuroblastoma and PC12 cell lines against certain stressors, while exhibiting neurotoxic qualities in a dose and time-dependent manner in certain hypothalamic neurons and causes grey matter reductions in primates and rodents.

Vucicevic et al.^9^ report that olanzapine initiates autophagy in SH-SY5Y blastoma cells. This present study demonstrates that olanzapine causes an increase in autophagosomes, lysosomes, LC3B-II and p62 proteins. Routinely, p62 is degraded upon autophagy completion; increased LC3B-II and p62, as found in this study, imply interference in the autophagy process. Haloperidol and clozapine are also reported to cause an increase of autophagosomes, lysosomes, LC3B-II and p62 proteins in primary rat neurons^22^. These APs are suggested to block the fusion between autophagosomes and lysosomes and thereby hinder the autophagy process. It is well known that aberrant autophagy may cause programmed cell death or apoptosis. Here, we confirm that olanzapine also inhibits the formation of autophagolysosomes in mHypoA-59 hypothalamic neurons and thereby causes cell death.

We demonstrate that olanzapine induces oxidative stress via increasing O_2_^−^; the most renowned free radical primarily produced in the mitochondria. Further analysis also reveals a substantial increase in mitochondrial mass, implying a defect in the mitophagy process. Vucicevic et al.^9^ also report that olanzapine (100 µM) causes oxidative stress and mitochondrial damage in SH-SY5Y cells after 24hrs. Another study treating human blood samples with 80 µM and 160 µM olanzapine for 1 hr found an increase in total oxidative stress at 160 µM. Although 100 µM was not measured, no oxidative stress was detected at concentrations lower than 80 µM^55^. Dietrich-Muszalska et al.^56^ conducted a similar study where blood samples treated with 20 and 40 µM olanzapine for 1 and 24hrs exhibit no significance in TBARS—an oxidative stress marker. These reports are consistent with our findings being that a concentration of ≥ 100 µM olanzapine causes oxidative stress while lower concentrations do not.

In the clinic olanzapine is reported to have antioxidant properties. For example, Al-Chalabi et al.^15^ report that administration of olanzapine 10–20 mg/day for 2 months significantly increases total antioxidant status and decreases MDA levels in the serum of schizophrenia patients. On the other hand, an animal study observing the effect of olanzapine (6 mM) on rat hepatocytes report cytotoxicity, increased ROS production, lipid peroxidation, mitochondrial and lysosomal damage after a mere 2 hr exposure period^57^. As previously suggested, it is possible that low doses of olanzapine (< 100 µM) have positive implications while, high doses (≥ 100 µM) may cause cellular damage via the ROS pathway. Also, ROS may be used as a biomarker to monitor possible olanzapine-induced toxic effects.

*N*-acetylcysteine is commonly used in the study of ROS and is now more often considered in psychiatric therapy. The aetiologies of psychiatric illnesses are multifactorial; this involves inflammatory pathways, oxidative stress, mitochondrial function and apoptotic pathways among others^27^. NAC is able to intervene in all of these pathways which is why it has gained popularity in the clinical setting. For example, in a double-blind, placebo-controlled, randomized trial of 140 participants, NAC was given in addition to regular medication for 6 months and an improvement in the Positive and Negative Symptoms Scale (PANSS) was observed^58^. In another case study, a treatment-resistant patient directed to hospital with a PANSS of 143 was treated with NAC for 7 days and her PANSS score decreased to 59^59^. In this current study we found that NAC reverses olanzapine-induced oxidative stress, mitochondrial damage and LC3B-II. Vucicevic et al.^9^ also report that NAC reversed olanzapine-induced LC3B conversion in SH-SY5Y cells. Given that NAC has beneficial effects in schizophrenia patients, it is possible that NAC administered in conjunction with olanzapine may alleviate olanzapine-induced effects via regulating ROS and mitophagy and reinforcing the stability of hypothalamic neurons.

## Supplementary information

Supplementary Information

## References

[1] Marston L, Nazareth I, Petersen I, Walters K, Osborn DPJ (2014). Prescribing of antipsychotics in UK primary care: a cohort study. BMJ Open.

[2] Turkheimer FE (2020). Normalizing the abnormal: do antipsychotic drugs push the cortex into an unsustainable metabolic envelope?. Schizophr. Bull..

[3] Moncrieff J, Leo J (2010). A systematic review of the effects of antipsychotic drugs on brain volume. Psychol. Med..

[4] Van Erp TG (2018). Cortical brain abnormalities in 4474 individuals with schizophrenia and 5098 control subjects via the enhancing neuro imaging genetics through meta analysis (ENIGMA) consortium. Biol. Psychiat..

[5] Haijma SV (2013). Brain volumes in schizophrenia: a meta-analysis in over 18,000 subjects. Schizophr. Bull..

[6] Huang XF, Song X (2019). Effects of antipsychotic drugs on neurites relevant to schizophrenia treatment. Med. Res. Rev..

[7] Vernon AC, Natesan S, Modo M, Kapur S (2011). Effect of chronic antipsychotic treatment on brain structure: a serial magnetic resonance imaging study with ex vivo and postmortem confirmation. Biol. Psychiat..

[8] Vernon AC (2014). Reduced cortical volume and elevated astrocyte density in rats chronically treated with antipsychotic drugs—linking magnetic resonance imaging findings to cellular pathology. Biol. Psychiat..

[9] Vucicevic L (2014). Autophagy inhibition uncovers the neurotoxic action of the antipsychotic drug olanzapine. Autophagy.

[10] 10Jevtić Dožudić, G. Z. *Mitohondrijalna disfunkcija u mozgu pacova perinatalno tretiranih fenciklidinom–Efekat antipsihotika*, Univerzitet u Beogradu-Medicinski fakultet, (2017).

[11] 11Drougard, A., Fournel, A., Valet, P. & Knauf, C. Impact of hypothalamic reactive oxygen species in the regulation of energy metabolism and food intake. *Front. Neurosci.* **9** (2015). 10.3389/fnins.2015.00056 PMC4338676 25759638

[12] Dröge W (2002). Free radicals in the physiological control of cell function. Physiol. Rev..

[13] Kim I, Rodriguez-Enriquez S, Lemasters JJ (2007). Minireview: selective degradation of mitochondria by mitophagy. Arch. Biochem. Biophys..

[14] Herken H (2000). Evidence that the activities of erythrocyte free radical scavenging enzymes and the products of lipid peroxidation are increased in different forms of schizophrenia. Mol. Psychiatry.

[15] Al-Chalabi BM, Thanoon IA, Ahmed FA (2009). Potential effect of olanzapine on total antioxidant status and lipid peroxidation in schizophrenic patients. Neuropsychobiology.

[16] Sagara Y (1998). Induction of reactive oxygen species in neurons by haloperidol. J. Neurochem..

[17] Walss-Bass C, Weintraub ST, Hatch J, Mintz J, Chaudhuri AR (2008). Clozapine causes oxidation of proteins involved in energy metabolism: a possible mechanism for antipsychotic-induced metabolic alterations. Int. J. Neuropsychopharmacol..

[18] Reinke A (2004). Haloperidol and clozapine, but not olanzapine, induces oxidative stress in rat brain. Neurosci. Lett..

[19] Merenlender-Wagner A (2015). Autophagy has a key role in the pathophysiology of schizophrenia. Mol. Psychiatry.

[20] Deheshi S, Pasqualotto BA, Rintoul GL (2013). Mitochondrial trafficking in neuropsychiatric diseases. Neurobiol. Disease.

[21] Gatliff J (2014). TSPO interacts with VDAC1 and triggers a ROS-mediated inhibition of mitochondrial quality control. Autophagy.

[22] Park J (2012). Haloperidol and clozapine block formation of autophagolysosomes in rat primary neurons. Neuroscience.

[23] Shin JH (2012). Sertindole, a potent antagonist at dopamine D 2 receptors, induces autophagy by increasing reactive oxygen species in SH-SY5Y neuroblastoma cells. Biol. Pharm. Bull..

[24] Vucicevic L, Misirkic-Marjanovic M, Harhaji-Trajkovic L, Maric N, Trajkovic V (2018). Mechanisms and therapeutic significance of autophagy modulation by antipsychotic drugs. Cell Stress.

[25] Baroncini M (2012). MRI atlas of the human hypothalamus. Neuroimage.

[26] Taylor D, McAskill R (2000). Atypical antipsychotics and weightgain—a systematic review. Acta Psychiatr. Scand..

[27] Dean O, Giorlando F, Berk M (2011). N-acetylcysteine in psychiatry: current therapeutic evidence and potential mechanisms of action. J. Psychiatry Neurosci.: JPN.

[28] Dhillon SS (2011). Cellular leptin resistance impairs the leptin-mediated suppression of neuropeptide Y secretion in hypothalamic neurons. Endocrinology.

[29] Kowalchuk C, Kanagasundaram P, McIntyre WB, Belsham DD, Hahn MK (2019). Direct effects of antipsychotic drugs on insulin, energy sensing and inflammatory pathways in hypothalamic mouse neurons. Psychoneuroendocrinology.

[30] Gozuacik D, Kimchi A (2007). Autophagy and cell death. Curr. Top. Dev. Biol..

[31] Lee J-A (2009). Autophagy in neurodegeneration: two sides of the same coin. BMB Rep..

[32] Okamoto A (2016). The antioxidant N-acetyl cysteine suppresses lidocaine-induced intracellular reactive oxygen species production and cell death in neuronal SH-SY5Y cells. BMC Anesthesiol..

[33] Martínez M-A (2020). Use of human neuroblastoma SH-SY5Y cells to evaluate glyphosate-induced effects on oxidative stress, neuronal development and cell death signaling pathways. Environ. Int..

[34] Han D (1997). Protection against glutamate-induced cytotoxicity in C6 glial cells by thiol antioxidants. Am. J. Physiol.-Regulat. Integrat. Comp. Physiol..

[35] Zhang F, Lau SS, Monks TJ (2010). The cytoprotective effect of N-acetyl-L-cysteine against ROS-induced cytotoxicity is independent of its ability to enhance glutathione synthesis. Toxicol. Sci..

[36] Yedjou CG, Tchounwou CK, Haile S, Edwards F, Tchounwou PB (2010). N-acetyl-cysteine protects against DNA damage associated with lead toxicity in HepG2 cells. Ethn. Dis..

[37] Spagnuolo G (2006). Effect of N-acetyl-L-cysteine on ROS production and cell death caused by HEMA in human primary gingival fibroblasts. Biomaterials.

[38] Citrome L, Kantrowitz JT (2009). Olanzapine dosing above the licensed range is more efficacious than lower doses: fact or fiction?. Expert Rev. Neurother..

[39] Qadri SF, Padala PR, Strunk JC, Boust SJ (2006). High-dose olanzapine orally disintegrating tablets for treatment-resistant psychosis. Prim. Care Compan. J. Clin. Psychiatry.

[40] Petersen AB, Andersen SE, Christensen M, Larsen HL (2014). Adverse effects associated with high-dose olanzapine therapy in patients admitted to inpatient psychiatric care. Clin. Toxicol..

[41] Robertson MD, McMullin MM (2000). Olanzapine concentrations in clinical serum and postmortem blood specimens - When does therapeutic become toxic?. J. Forensic Sci..

[42] Hiemke C (2018). Consensus guidelines for therapeutic drug monitoring in neuropsychopharmacology: update 2017. Pharmacopsychiatry.

[43] Rao ML, Hiemke C, Grasmader K, Baumann P (2001). Olanzapine: pharmacology, pharmacokinetics and therapeutic drug monitoring. Fortschr. Neurol. Psychiatr..

[44] Olesen OV, Linnet K (1999). Olanzapine serum concentrations in psychiatric patients given standard doses: the influence of comedication. Ther. Drug Monit..

[45] Aravagiri M, Teper Y, Marder SR (1999). Pharmacokinetics and tissue distribution of olanzapine in rats. Biopharm. Drug Dispos..

[46] Kornhuber J (1999). Persistence of haloperidol in human brain tissue. Am. J. Psychiatry.

[47] Anwar IJ, Miyata K, Zsombok A (2016). Brain stem as a target site for the metabolic side effects of olanzapine. J. Neurophysiol..

[48] Abekawa T, Ito K, Nakagawa S, Nakato Y, Koyama T (2008). Olanzapine and risperidone block a high dose of methamphetamine-induced schizophrenia-like behavioral abnormalities and accompanied apoptosis in the medial prefrontal cortex. Schizophr Res..

[49] Lu XH, Bradley RJ, Dwyer DS (2004). Olanzapine produces trophic effects in vitro and stimulates phosphorylation of Akt/PKB, ERK1/2, and the mitogen-activated protein kinase p38. Brain Res..

[50] Wei Z, Bai O, Richardson JS, Mousseau DD, Li XM (2003). Olanzapine protects PC12 cells from oxidative stress induced by hydrogen peroxide. J. Neurosci. Res..

[51] Li XM, Xu H (2007). Evidence for neuroprotective effects of antipsychotic drugs: implications for the pathophysiology and treatment of schizophrenia. Int. Rev. Neurobiol..

[52] Nazarians-Armavil A, Menchella JA, Belsham DD (2013). Cellular insulin resistance disrupts leptin-mediated control of neuronal signaling and transcription. Mol. Endocrinol..

[53] Dorph-Petersen KA (2005). The influence of chronic exposure to antipsychotic medications on brain size before and after tissue fixation: a comparison of haloperidol and olanzapine in macaque monkeys. Neuropsychopharmacol.: Off. Publ. Am. College Neuropsychopharmacol..

[54] Konopaske GT (2007). Effect of chronic exposure to antipsychotic medication on cell numbers in the parietal cortex of macaque monkeys. Neuropsychopharmacol.: Off. Publ. Am. College Neuropsychopharmacol..

[55] Türkez H, Toğar B (2010). The genotoxic and oxidative damage potential of olanzapine in vitro. Toxicol. Ind. Health.

[56] Dietrich-Muszalska A, Kontek B, Rabe-Jablonska J (2011). Quetiapine, olanzapine and haloperidol affect human plasma lipid peroxidation in vitro. Neuropsychobiology.

[57] Eftekhari A, Azarmi Y, Parvizpur A, Eghbal MA (2016). Involvement of oxidative stress and mitochondrial/lysosomal cross-talk in olanzapine cytotoxicity in freshly isolated rat hepatocytes. Xenobiotica.

[58] Berk M (2008). N-acetyl cysteine as a glutathione precursor for schizophrenia–a double-blind, randomized, placebo-controlled trial. Biol. Psychiat..

[59] Bulut M, Savas HA, Altindag A, Virit O, Dalkilic A (2009). Beneficial effects of N-acetylcysteine in treatment resistant schizophrenia. World J. Biol. Psychiatry.

