# Supplementary material for: N-acetylcysteine prevents olanzapine-induced oxidative stress in mHypoA-59 hypothalamic neurons
Source: Sci Rep. 2020 Nov 5;10:19185. doi: 10.1038/s41598-020-75356-3 (PMC7644715; doi:10.1038/s41598-020-75356-3)
Supplement: Supplementary file 1 — Supplementary Information [file 41598_2020_75356_MOESM1_ESM.pptx]

## Slide 1
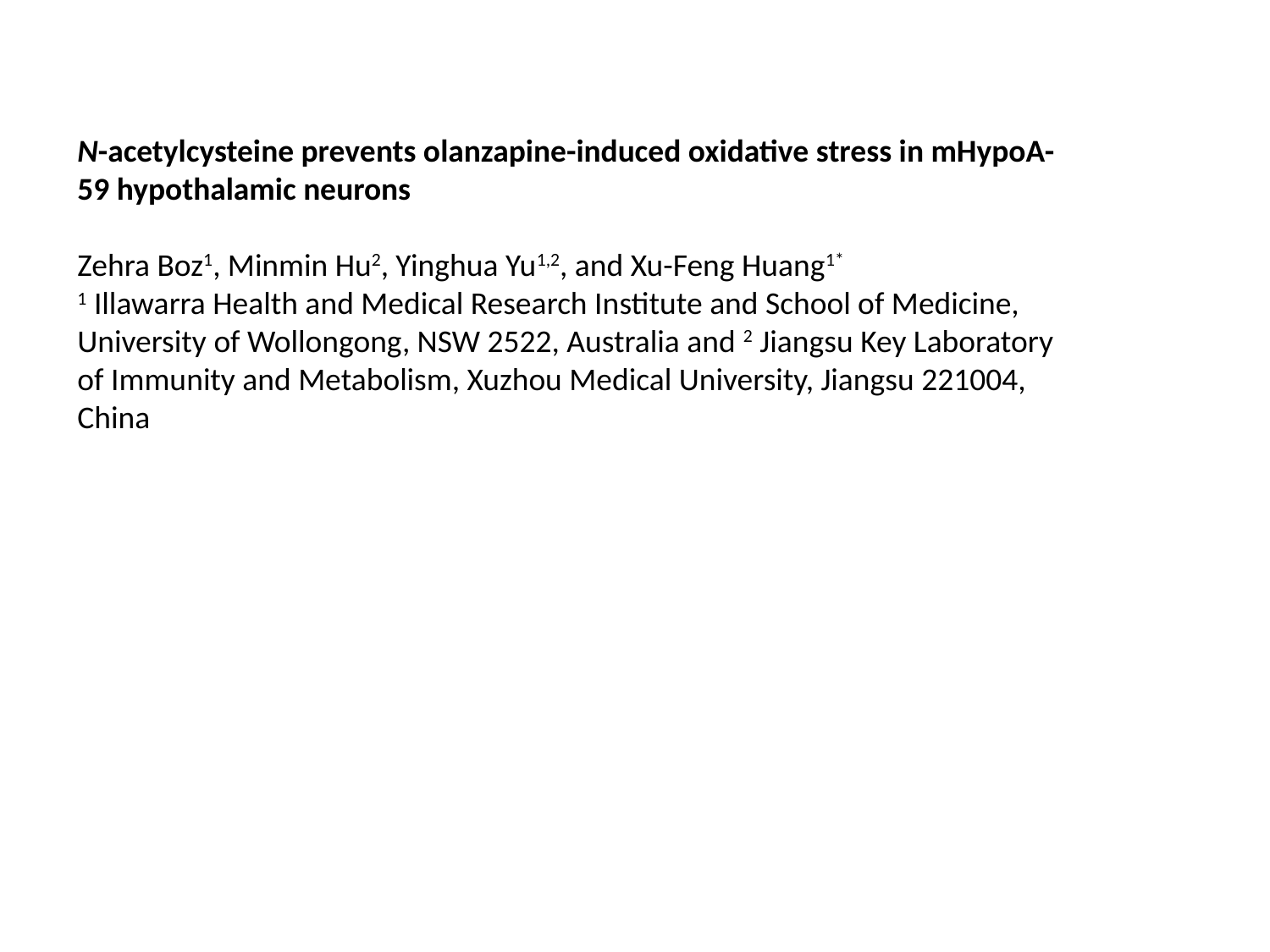

N-acetylcysteine prevents olanzapine-induced oxidative stress in mHypoA-59 hypothalamic neurons
Zehra Boz1, Minmin Hu2, Yinghua Yu1,2, and Xu-Feng Huang1*
1 Illawarra Health and Medical Research Institute and School of Medicine, University of Wollongong, NSW 2522, Australia and 2 Jiangsu Key Laboratory of Immunity and Metabolism, Xuzhou Medical University, Jiangsu 221004, China

## Slide 2
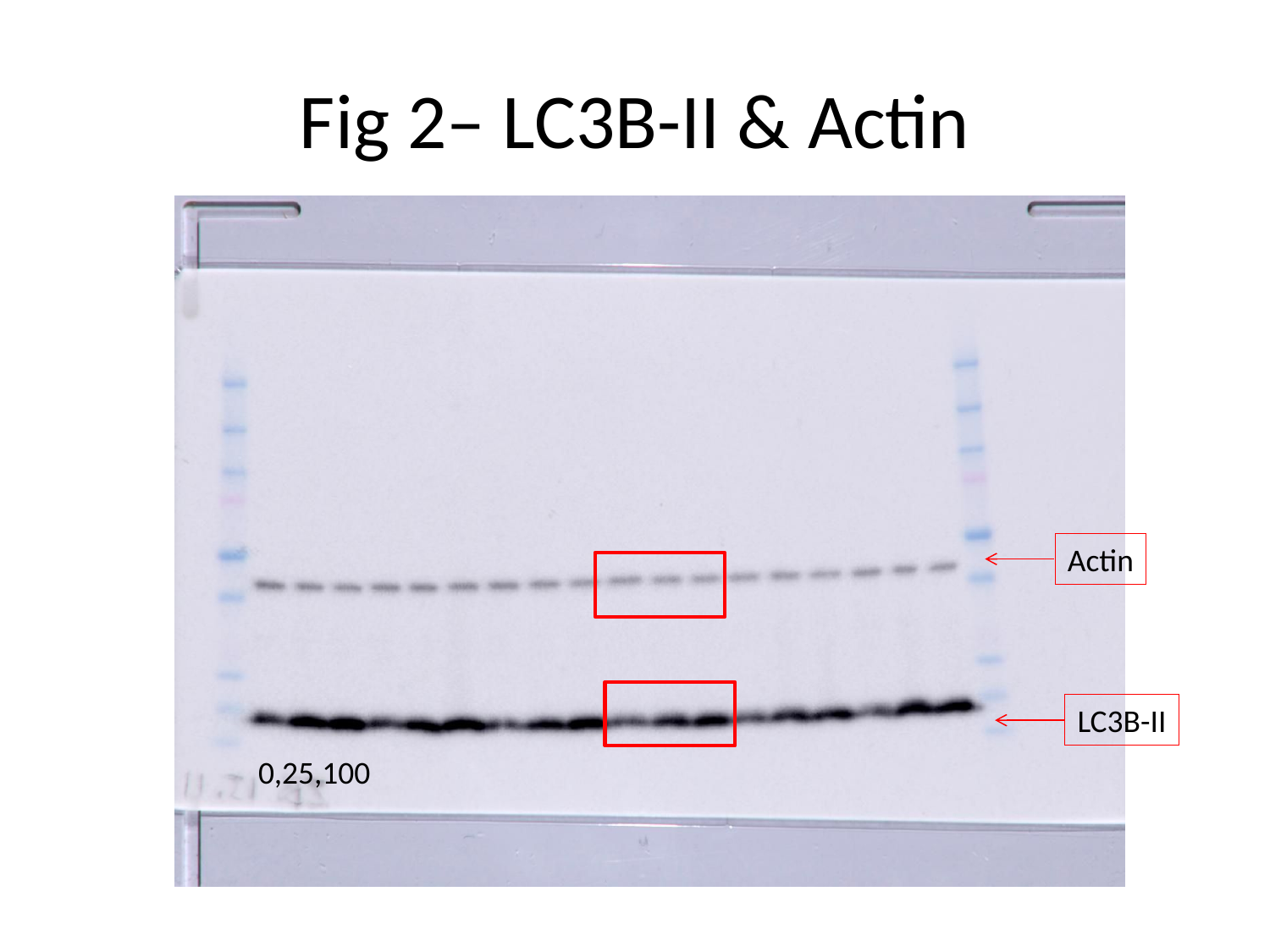

# Fig 2– LC3B-II & Actin
Actin
LC3B-II
0,25,100

## Slide 3
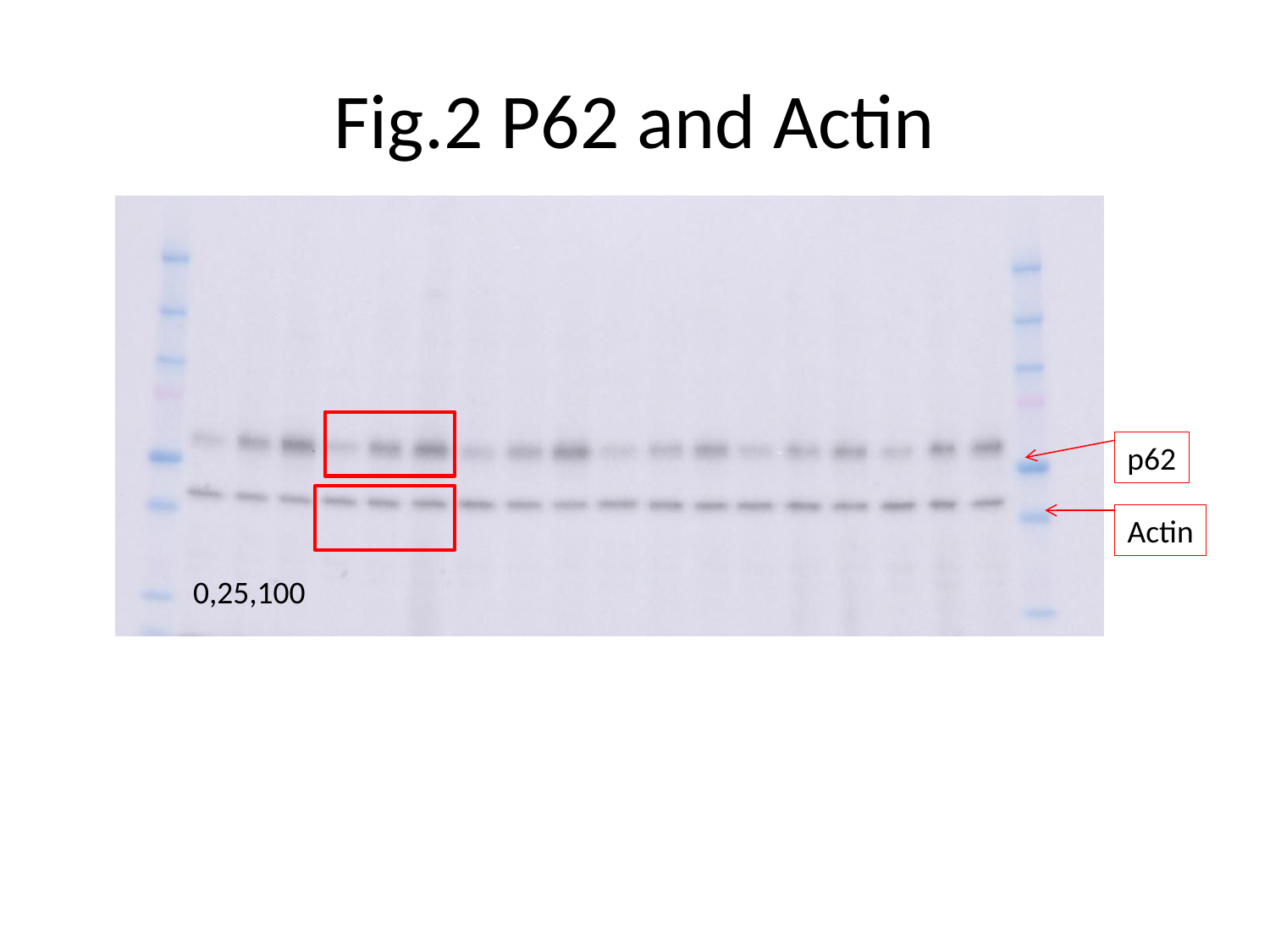

# Fig.2 P62 and Actin
p62
Actin
0,25,100

## Slide 4
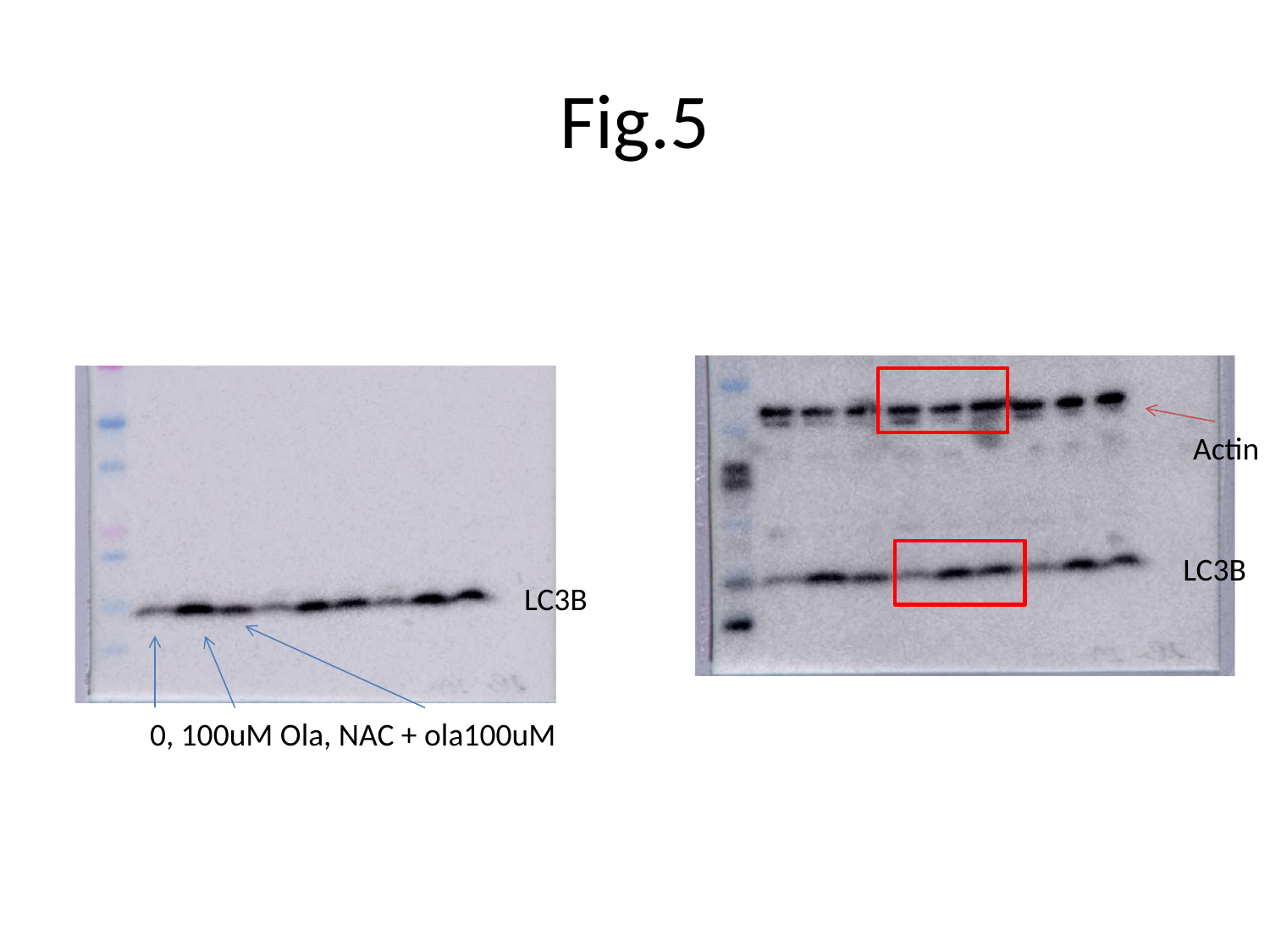

# Fig.5
Actin
LC3B
LC3B
0, 100uM Ola, NAC + ola100uM
